# Accuracy and Completeness of Long Read Metagenomic Assemblies

**DOI:** 10.3390/microorganisms11010096

**Published:** 2022-12-30

**Authors:** Jeremy Buttler, Devin M. Drown

**Affiliations:** 1 Department of Biology and Wildlife, University of Alaska Fairbanks, Fairbanks, AK 99775, USA; 2 Institute of Arctic Biology, University of Alaska Fairbanks, Fairbanks, AK 99775, USA

**Keywords:** nanopore sequencing, benchmarking, microbial communities, long read assemblers

## Abstract

Microbes influence the surrounding environment and contribute to human health. Metagenomics can be used as a tool to explore the interactions between microbes. Metagenomic assemblies built using long read nanopore data depend on the read level accuracy. The read level accuracy of nanopore sequencing has made dramatic improvements over the past several years. However, we do not know if the increased read level accuracy allows for faster assemblers to make as accurate metagenomic assemblies as slower assemblers. Here, we present the results of a benchmarking study comparing three commonly used long read assemblers, Flye, Raven, and Redbean. We used a prepared DNA standard of seven bacteria as our input community. We prepared a sequencing library using a VolTRAX V2 and sequenced using a MinION mk1b. We basecalled with Guppy v5.0.7 using the super-accuracy model. We found that increasing read depth benefited each of the assemblers, and nearly complete community member chromosomes were assembled with as little as 10× read depth. Polishing assemblies using Medaka had a predictable improvement in quality. We found Flye to be the most robust across taxa and was the most effective assembler for recovering plasmids. Based on Flye’s consistency for chromosomes and increased effectiveness at assembling plasmids, we would recommend using Flye in future metagenomic studies.

## 1. Introduction

Current methods for identifying microbes involve isolating and sequencing individual community members, amplicon sequencing 16S rRNA genes, or metagenomics [1,2]. Isolating individual microbes requires culturing, which is often difficult or practically impossible [1]. Sequencing 16S rRNA genes cannot provide information on the entire genomes, such as genes that might increase virulence or provide antibiotic resistance [2]. Metagenomics is a method where an entire sample is sequenced, and the individual community members are sorted out later with bioinformatic analyses [3]. Metagenomic sequencing can detect unculturable and novel community members [1]. The individual community member sequences can be studied to identify pathogens in difficult to diagnose disease, genes that may increase virulence, and look for correlations between co-infecting pathogens that increase disease severity [1,2,4,5]. Currently, most metagenomic approaches use Illumina based technology, which produces high accuracy, short reads [2]. Over the past several years, Oxford Nanopore Technologies (ONT) has increased sequencing throughput and yield to be reasonable for metagenomic studies. While these reads are error prone, the reads are also orders of magnitude longer than short read platforms [2].

The short reads (150–300 bp) from Illumina sequencing make genome assembly difficult for complex communities. Short read lengths do not facilitate scaffolding multiple contigs built for a genome into a single scaffold, resulting in fragmented assemblies [6]. Short reads cannot span long repeat regions, causing repeat regions to collapse, providing less complete assemblies [7]. More complete genomes can be assembled using long read sequencing technologies, such as ONT or PacBio [6]. ONT sequencers platforms (e.g., MinION) have produced reads greater than 2 mb long and can easily produce libraries with mean read lengths greater than 16 kb, which makes it possible to assemble long repeat regions [8,9,10]. However, the high error rates of nanopore sequencing also prevent short read assemblers from producing quality assemblies with long read data [10,11].

Three commonly used long read specific assemblers include Flye, Raven, and Redbean [11,12,13,14]. Flye is a long read metagenomic assembler that constructs a repeat graph to assemble and polish contigs [15,16]. These contigs are then used to build an assembly graph with A-Bruijn [15,16]. Previous studies found that while Flye can build more accurate metagenomic assemblies than Raven or Redbean, it also takes more time and memory [11,17]. Raven is a fast assembler that uses an Overlap-Layout-Consensus (OLC) approach to build an assembly graph from raw reads [18]. For some individual assemblies Raven can have comparable accuracy to Flye after the assemblies are polished, but has less accuracy for metagenomic assemblies [13,17,18]. Redbean is another fast assembler that follows the OLC concept by using a fuzzy de Bruijn graph to build assemblies from raw reads [19,20]. Previous studies have found that Redbean uses more memory and builds less accurate assemblies than Raven [11,17].

Benchmarking is used to compare bioinformatics tools and to determine which tool is best suited for a particular task [12,21]. Benchmarking studies for metagenomic assemblers often include well characterized communities or mock communities, like one of the many ZymoBIOMICS Microbial Community Standards [6,11,15,22]. Mock communities are synthetic communities composed of multiple known microbes, with known sequences and abundances [23]. This information allows for accurate assessment and comparison of assemblers for metagenomic data.

A past benchmarking study using a ZymoBIOMICS Microbial Community Standard found that Raven and Redbean could not build complete assemblies for the *E. coli* and *Salmonella enterica* community members [11]. Raven did well for the other community members in the ZymoBIOMICS Microbial Community Standard [11] Raven also performs well for individual assemblies of *E. coli* [13,14]. These differences in performance suggest that the high read error rate may cause Raven to confuse genome fragments from other community members with *E. coli* fragments. If so, a higher read accuracy, as produced by Guppy 5.0.7, may allow Raven to assemble all community members from the mock community with similar accuracy to Flye. Another weakness of Raven and Redbean, is that they often fail to build assemblies for plasmids [17]. These weaknesses may limit the performance of Raven and Redbean for complex metagenomic assemblies, where plasmids may be common and particular community members may be present.

Improvements in converting the electrical signal from nanopore sequencing to to nucleotides (basecalling) have led to increased read level nanopore sequence accuracy. The release of new models (e.g., super-accuracy model) for Guppy have pushed modal accuracy even higher (see also https://nanoporetech.com/accuracy (accessed on 18 July 2022)). As the individual reads improve in quality, faster assemblers, such as Raven, may be able to build assemblies of problematic community members, such as *E. coli*, with comparable accuracy to slower, but more accurate assemblers, like Flye.

Here, we compare the completeness and accuracy of metagenomic assemblies built with Flye, Raven, and Redbean. We used data basecalled with the super-accuracy model of Guppy to systematically explore the impact of read depth. From this comparison, we contrast the areas of strength and weakness of long read metagenomic assemblers.

## 2. Materials and Methods

### 2.1. Sequencing

We sequenced a mock community standard (ZymoBIOMICS HMW DNA Standard, catalog #D6322) using long read sequencing to compare metagenomic assembly methods. The HMW DNA standard is a synthetic microbial community comprising three Gram negative bacteria, four Gram positive bacteria, and one yeast (Table 1). Bacterial community members have a genome size between 2.73 mb to 6.792 mb, a GC content between 32.9% and 66.2% (Table 1). The *E. coli* plasmid is 110,009 bp, the *S. aureus* 1 plasmid is 6339 bp, the *S. aureus* 2 plasmid is 2218 bp, and the *S. aureus* 3 plasmid is 2995 bp. Each bacterial community also contributed 14% of nucleotides in the mock community (Table 1). We excluded the *Saccharomyces* community member due to the reference genome being highly fragmented. The template DNA in the community has a mean length of 24 kb. Reference genome sequences can be found at https://s3.amazonaws.com/zymo-files/BioPool/D6322.refseq.zip (accessed on 15 July 2021).

We used 1 μg of the HMW DNA standard as input for the VolTRAX V2 (ONT) to prepare a sequencing library (VSK-VSK002 workflow). The VolTRAX library is analogous to the Rapid Sequencing library and results in additional DNA template fragmentation as the library is prepared. We sequenced the prepared library using the MinION mk1b (ONT) on a r9.4.1 flow cell (FLO-MIN106) for 48 h (VSK002 script). We basecalled the reads using Guppy version 5.0.7 with the super-accuracy model (-c dna_r9.4.1_450bps_sup.cfg). We set a minimum quality filter of ≥ 10 (-min_qscore 10).

To generate a subsample of reads, we used trycycler [24]. We used a genome size of 42 mb and the –min read depth parameter to generate subsamples of 420 mb, 840 mb, 1260 mb, 2100 mb, 4200 mb, and 8400 mb. These total yields should theoretically represent 10×, 20×, 30×, 50×, 100×, and 200× read depths. At each read depth, we produced 12 subsamples for a total of 72 datasets. The mean number of bases, mean longest read length, and mean N50 for each read depth was found using NanoStat –fastq [25].

### 2.2. Assembly and Polishing

For this comparison, we used three commonly used assemblers to construct metagenomic assemblies of our data sets, Flye, Redbean, and Raven. We used metaFlye (Flye –meta) version v2.8.3 [15] with default parameters specifying nanopore reads (–nano-raw) and the following options in recover plasmids (–plasmids) and metagenomes (–meta). We used Raven v1.5.1 [18] with default parameters. We used Redbean v2.5 [19] with default parameters specifying nanopore reads (-x ont), and a genome size of 42 mbases (-g 42m).

We polished all assemblies using one round of Racon v1.4.22 [26] followed by one round of Medaka v1.4.3 (https://github.com/nanoporetech/medaka, accessed on 15 July 2021), specifying the super-accuracy model (-m r941_min_sup_g507). For Racon, we used the ONT suggested parameters: score for matching bases (-m 8), score for mismatching bases (-x -6), gap penalty (-g -8), window size (-w 500), and mean quality threshold for each window (-q -1).

### 2.3. Quality Assessment

We measured assembly quality and completeness with the genome fraction output by MetaQuast v5.1.0 [27]. For MetaQuast we used the references in (Table 1) to measure the completeness of both the polished and unpolished metagenomic assemblies. We measured assembly accuracy with the median Q-score output by Pomoxis assess_assembly https://github.com/nanoporetech/pomoxis/ (accessed on 15 July 2021). Pomoxis was used with the references in (Table 1) to find the quality scores (Q-scores) of the assemblies. For each assembly, we calculated Q-scores for chromosomes and plasmids separately.

We completed all analysis, including assembly, polishing, and assembly quality assessment on a server with an Intel Core i9 9900K 3.6 GHz Eight Core (16 thread) CPU, a Nvidia Quadro GV100 GPU, and 128 GB of ram. We measured the time required and the max memory used to build each assembly using GNU time with parameter -f %ee. The time, assembly, polishing, MetaQuast, and Pomoxis steps were automated using custom bash scripts https://github.com/jeremyButtler/assembler-scripts (accessed on 22 June 2022).

We used R v4.1.1.1 [28] with ggplot2 [29], cowplot [30], ggpubr [31], tidyr [32], data.table [33], stringr [34], and RColorBrewer [35] to build graphs for the metagenome fraction, genome fraction, median Q-scores, number of misassemblies, time, and maximum memory usage. The metagenome fraction was found by dividing the number of bases that were aligned to a community member in a replicate by the total bases in the community.

## 3. Results

### 3.1. Subsampling Statistics

We sequenced the ZymoBIOMICS HMW DNA Standard on a nanopore sequencer and subsampled reads into subsamples of 420 mb (~10× read depth), 840 mb (~20× read depth), 1260 mb (~30× read depth), 2100 mb (~50× read depth), 4200 mb (~100× read depth), and 8400 mb (~200× read depth). For each targeted read depth, our mean number of bases was very close to our target number of base pairs (Table 2). The mean read N50 between our read depths differed by only 18 base pairs (15,012 to 15,030 bp) and was 300 bp greater than our raw data mean read N50 of 14,703 bp (Table 2). Each time the read depth was doubled, we saw a two-fold increase in the mean number of reads (Table 2). Our mean Q-score for the raw data was 13.5.

### 3.2. Chromosome

#### 3.2.1. Genome Fraction

**Figure 1:**
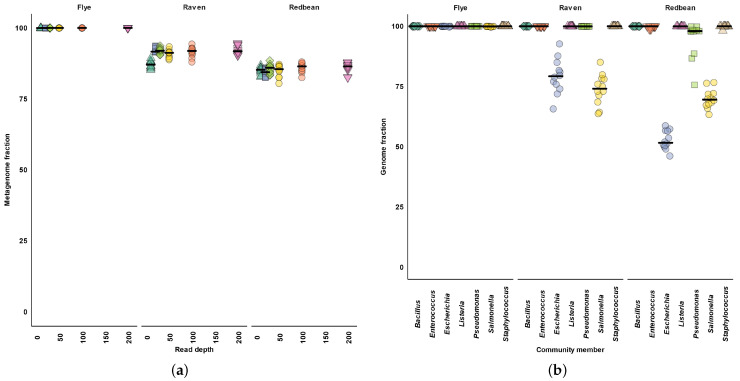
Chromosome completeness. (**a**) Metagenome fraction, percentage of aligned bases to the metacommunity reference genomes in a replicate. (**b**) Genome fraction of each community member with 200× read depth. Color and shape indicate different community members. Horizontal bars indicate the medians across subsamples.

Across all read depths, we found Flye produced assemblies that aligned with nearly 100% of the community of reference genomes (metagenome fractions) (Figure 1a). Even at our smallest read depth of 10×, Flye recovered nearly 100% of the metagenomic fraction (Figure 1a). With increasing read depth, Raven and Redbean produced assemblies with improved metagenome fractions (Figure 1a). Raven and Redbean reached a maximum metagenome fraction of 95% at 200× read depth (Figure 1b). At the individual community member level, Flye, Raven, and Redbean produced assemblies with over 99.9% median genome fractions for most of the community members (Figure 1, Table A1). With Raven and Redbean having the most difficulty assembling *Escherichia coli* and *Salmonella enterica*, recovering less than 80% genome fraction even at 200× read depth (Figure 1b).

#### 3.2.2. Accuracy (Q-Score)

**Figure 2:**
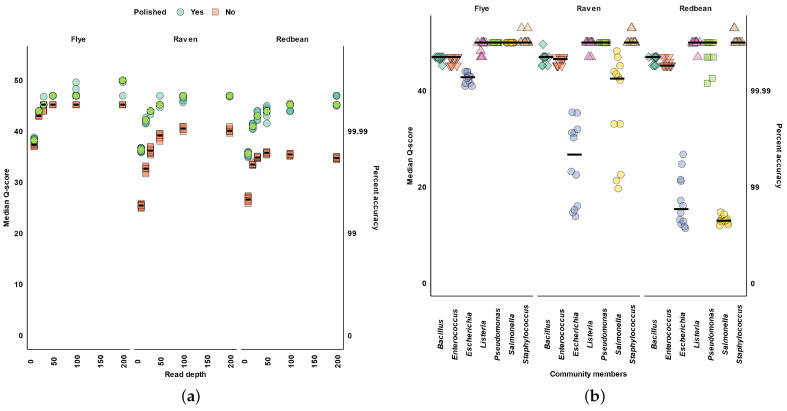
Chromosome accuracy. (**a**) Median quality (Q-score) for chromosomes before (red/orange squares) and after polishing (blue/green circles). (**b**) Individual community member median Q-score at 200× read depth. Horizontal bars indicate the median across subsamples. Color and shape indicate different community members (**b**).

Across all read depths, we found Flye produced the most accurate metagenomic assemblies, followed by Raven, and then Redbean (Figure 2a). Increased read depth and polishing, predictably improved the median quality scores (Q-scores) of assemblies from all assemblers (Figure 2a). All assemblers had a large improvement in Q-scores between 10× and 50× read depth (Figure 2a). At 200× read depth Flye reached a maximum Q-score of 50, while Raven and Redbean reached a maximum Q-score of 46 and 45, respectively, (Figure 2a).

**Figure A1:**
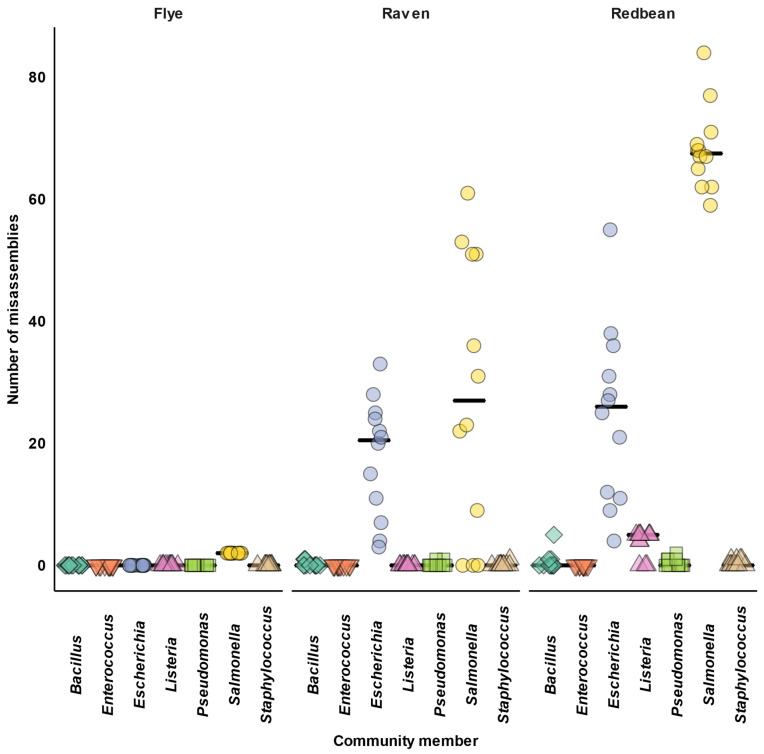
Chromosome misassemblies at 200× read depth. Horizontal bars indicate the median value across replicate samples.

At the individual community member level, Raven and Redbean had the most difficulty in the assembly of *E. coli* and *S. enterica* (Figure 2b). *E. coli* assemblies produced with Flye were more accurate (median Q-score 42.81) than those from Raven (26.73) and Redbean (under 20) (Figure 2b). *S. enterica* assemblies produced by Flye were highly accurate (median Q-score 50), while Raven was slightly less accurate (42.54), but Redbean produced error prone assemblies (under 20) (Figure 2b). We also found that Raven and Redbean, but not Flye, had over 10 misassemblies for *E. coli* and *S. enterica* (Figure A1).

### 3.3. Plasmids

#### 3.3.1. Genome Fraction

**Figure 3:**
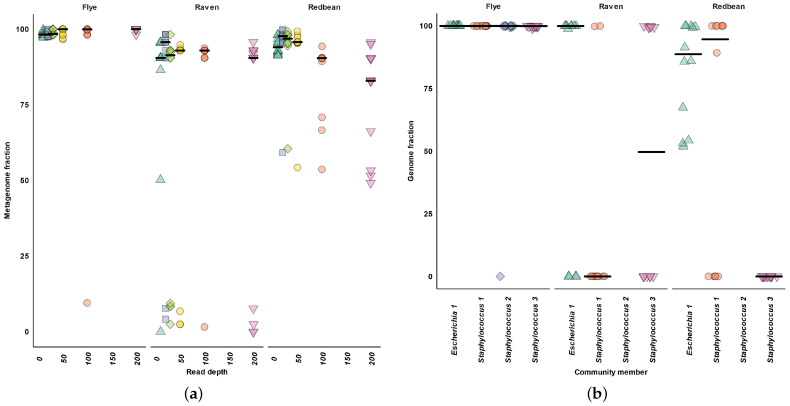
Plasmid completeness. (**a**) Plasmid metagenome fraction. (**b**) Plasmid genome fraction at 200× read depth. The horizontal bars indicates the median.

Across all read depths, we found Flye recovered over 94% of the plasmid genomes (Figure 3a). After 50× read depth Flye recovered nearly 100% of the plasmid genomes (Figure 3a). After 20× read depth, Raven and Redbean decreased the recovery of plasmid genomes (Figure 3a). Raven and Redbean assembled a maximum of 95% of the plasmid genome at 20× read depth (Figure 3a).

**Figure A2:**
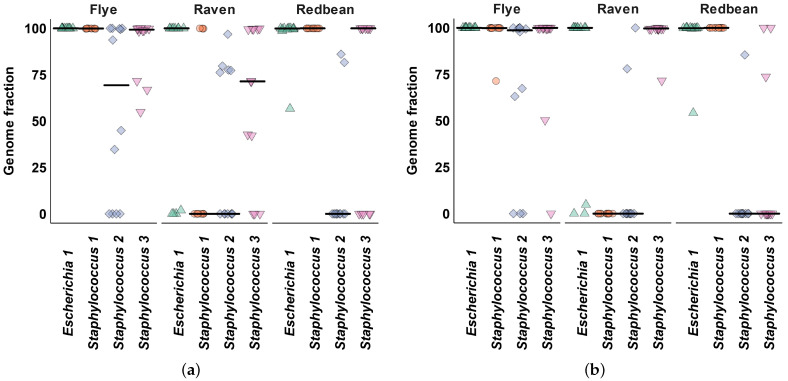
30× and 50× plasmid completeness. Horizontal bars indicate the median across replicates. (**a**) 30× read depth. (**b**) 50× read depth. *E. coli* is 110009 bases, *S. aureus* 1 is 6339 bases, *S. aureus* 2 is 2218 bases, and *S. aureus* 3 is 2995 bases long.

At the individual plasmid level, Raven and Redbean both struggled with the plasmids smaller than 7 kb (Figure 3b). Raven and Redbean assembled more of plasmids under 7 kb at 30× and 50× read depth than at 200× read depth (Figure A2a,b). Raven could assemble the 2995 bp plasmid for all replicates at 50× read depth, but not at 200× read depth (Figure A2b).

#### 3.3.2. Accuracy (Q-Score)

**Figure 4:**
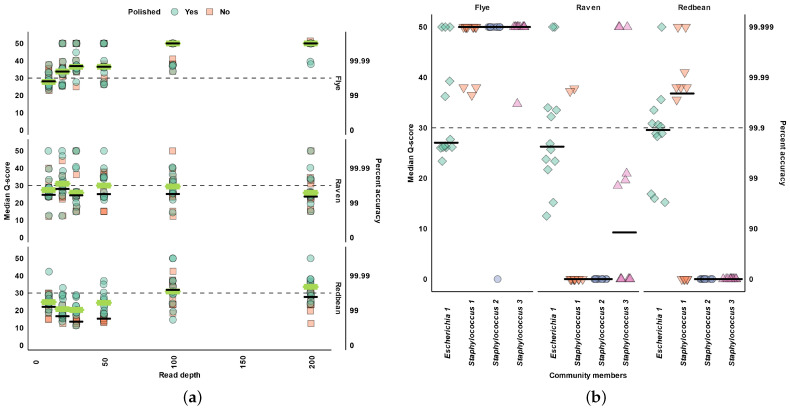
Plasmid accuracy. (**a**) Median Q-score for all replicates at 10×, 20×, 30×, 50×, 100×, and 200× read depth. (**b**) Median Q-score for plasmids at 200× read depth. Horizontal bars indicate the median. The dashed line indicates the highest Q-score for Raven.

Across all read depths, we found that found that Flye assembled the most accurate plasmids (Figure 4a). With increased read depth, Flye produced more accurate plasmid assemblies (Figure 4a). However, Polishing did not improve the accuracy of Flye plasmid assemblies (Figure 4a). At 100× read depth, Flye plasmid assemblies had a median Q-score of 50 (Figure 4a).

Across all read depths, polishing Raven and Redbean plasmid assemblies resulted in more accurate plasmid genomes (Figure 4a). Increased read depth did not imporeve the accuracy of Raven produced plasmid assemblies (Figure 4a). Beyond 50× read depth, Redbean produced more accurate plasmid assemblies than Raven (Figure 4a). However, Raven build more accurate plasmids assemblies than Redbean when the read depth was under 100× (Figure 4a).

**Figure A3:**
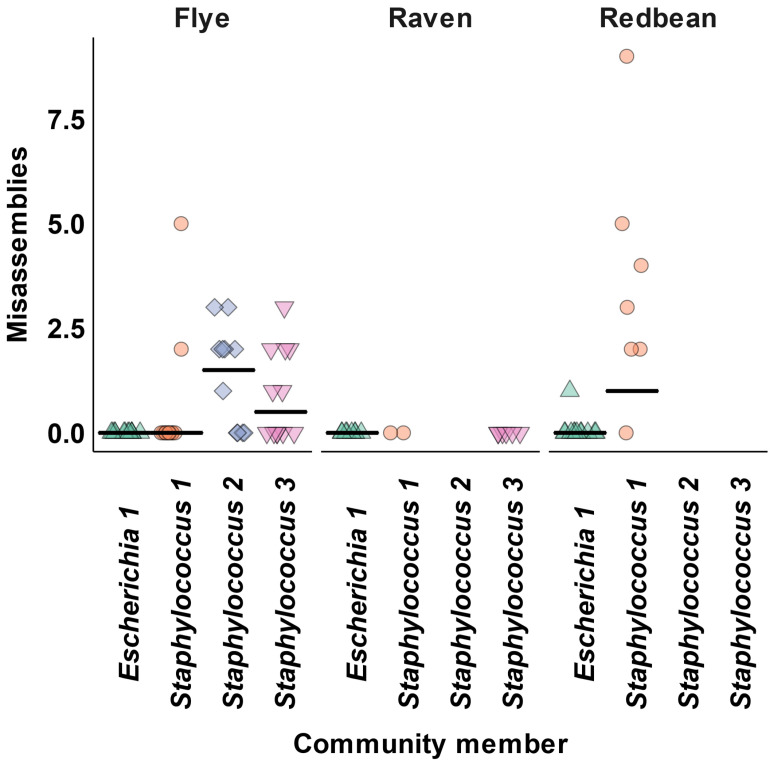
Plasmid misassemblies at 200× read depth. Horizontal bars indicate the median value across replicate samples.

At the individual plasmid level, only the *E. coli* 110,009 bp plasmid could be assembled by all assemblers (Figure 4b). All assemblers had a similar accuracy for the *E. coli* plasmid (Q-scores around 26) (Figure 4b). All assemblers were able to assemble the *E. coli* plasmid without misassemblies, but Flye and Redbean did have misassemblies for the plasmids under 7 kb (Figure A3). However, Flye assembled almost all replicates for each plasmid and had near perfect median Q-scores for plasmids under 7 kb (Figure 4b).

### 3.4. Assembly Time and Memory Usage

**Figure 5:**
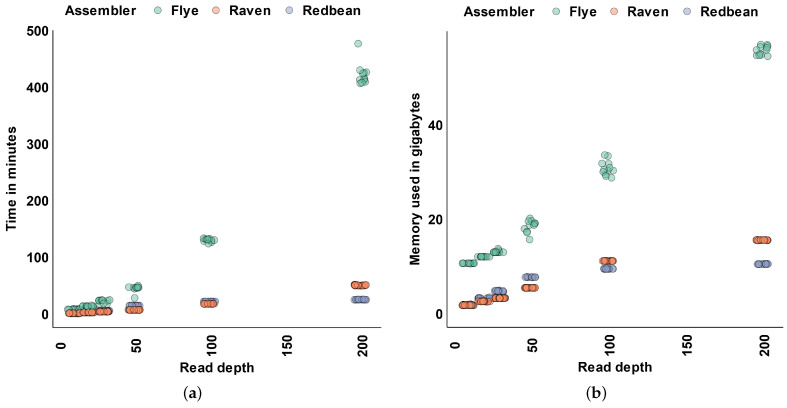
Time and memory usage of each assembler. (**a**) Time usage. (**b**) Memory usage.

Predictably, we found that assemblers needed more time and memory to build an assembly with greater read input (Figure 5a,b). When the read depth was under 50×, all assemblers used less than 30 min to complete an assembly (Figure 5a). At 200× read depth, Flye needed over 400 minutes to complete an assembly (Figure 5a). With that same input Raven required just 50 min and Redbean required only 25 minutes to complete an assembly (Figure 5a).

Across all read depths, Raven and Redbean used less memory than Flye to build an assembly (Figure 5b). At read depths under 100×, Raven used less memory than Redbean to build an assembly (Figure 5b). At 50× read depth, Raven needed 5.5 Gb of memory to build an assembly, while Redbean needed 7.7 Gb of memory to build an assembly (Figure 5b). Beyond 50× read depth, Raven used more memory than Redbean to build an assembly (Figure 5b). At 200× read depth, Raven used 15.6 Gb of memory to build an assembly, while Redbean used 10.5 Gb of memory to build an assembly (Figure 5b). Flye used the most memory to build an assembly, requiring 10.6 Gb of memory at 10× read depth and 55.8 Gb of memory at 200× read depth (Figure 5b).

## 4. Discussion

We compared the accuracy and completeness of metagenomic assemblies built by three long read assemblers, Flye, Raven, and Redbean. For chromosomes, we found Flye was the only assembler that made near complete and accurate genomes for all community members. For plasmids, we found Flye was the only assembler that could assemble all plasmids reliably. However, Raven and Redbean were superior to Flye in time and memory usage.

### 4.1. Effect of Read Depth

For chromosomes, we found with increased read depth, all assemblers made more accurate and complete assemblies. We found that there was a sharp increase in accuracy between 10× and 50× read depth. At 10× read depth, Flye was the only assembler that had near complete metagenome fractions. Showing that Flye should be used for low read depth datasets. However, for more accurate assemblies, future metagenomic studies should continue to aim for a read depth of at least 30×.

For plasmids, we found most plasmids under 7 kb were assembled best by Flye, with the most plasmids recovered at 200× read depth. However, Raven and Redbean had decreased small plasmid recovery at deeper read depths and performed best at read depths between 20× or 50×. The decrease in assembled plasmids under 7 kb at deeper read depths suggests that Raven and Redbean are discarding smaller reads and contigs at deeper read depths. This results in plasmids under 7 kb being missed at deeper read depths but being retained at more shallow read depths. These observations are consistent with Wick and Holt [17], who also found that both Raven and Redbean struggled to complete assemblies of smaller plasmids. These results highlight the weakness in Raven and Redbean for recovering plasmids.

We found that the accuracy of the larger *E. coli* plasmid (Q-score under 30) was much lower than the chromosome assemblies (40 or 50). This suggests that the plasmids have more error prone regions, assemblers are more likely to make misassemblies for plasmids, or that the plasmid references have more errors than the chromosome references. For reference errors, Flye could often assemble plasmids under 3 kb with no indels or mismatches and with only 2 to 3 misassemblies. Errors in the references are a less likely but still a potential explanation for why Flye, Raven, and Redbean had poor performance for the *E. coli* plasmid.

For misassembly errors, we found all assemblers had no errors in *E. coli* plasmid assemblies at 200× read depth, showing that the problem is not from misassemblies in the *E. coli* plasmid. Other sources of errors in the *E. coli* palsmid could be from more error prone regions in the *E. coli* plasmid or errors inserted by the assemblers in the assembly of the *E. coli* plasmid. During the process of generating these results, a new version of Flye was released (v2.9), which included improvements for recovering plasmids and accounts for the improved accuracy of the super-accuracy model. However, more testing with a broader range of plasmid sizes is needed to determine if the errors are from error prone regions or from the assembler.

### 4.2. Metagenomics and Viruses

Though our study examined a mock microbial community mostly consisting of bacterial genomes, our results still provide insights into how reliable each assembler may be for viral metagenomic assemblies. The *E. coli* plasmid in our study is 110 kb long, which is close to or under the size of a large virus, such as the 170 to 190 kb African swine fever virus (ASFV) [36]. We have previously used Flye to recover ASFV successfully from a metagenomic sample [37], while the smaller plasmids in our study are near the size of small viruses, such as porcine circovirus type 2, which is 1.76 kb long [38].

For the larger plasmids and likely larger viruses, we found that Raven or Redbean would likely work as well as Flye. However, only Flye could make reliable assemblies for the smaller plasmids and so, is the only reliable assembler for smaller viruses. Even then Flye will often have a few misassemblies, so it might be best to use an assembler, like viralFlye that is designed for viruses [39]. However, viralFlye is specialized for virus detection and thus has limitations on the max genome size [39]. This may limit viralFlye’s use for bacterial community members. Making Flye or assemblies made with both viralFlye and Flye the best option for sequencing mixed communities of viruses and bacteria.

### 4.3. Effect of Polishing

We found that polishing improved the accuracy of all chromosome assemblies. However, for Flye and Redbean, polishing continued to improve the accuracy at 200× read depth. This suggests that even more data will improve the accuracy of polished Flye assemblies. To achieve highly accurate assemblies, we would recommend polishing and using the greatest read depth as possible.

For Flye, polishing had little effect on the accuracy of plasmid assemblies. Instead, most plasmids smaller than 3 kb had no indels or mismatches at 200× read depth. This shows that polishing did not decrease the accuracy of the perfect assemblies. Likely, the high accuracy was due to the genome sizes of the plasmids being smaller than the error rate of consensuses assemblies (one error in 10,000 bp for chromosomes). The idea of size is somewhat supported by the ten fold larger *E. coli* plasmid assemblies built by Flye having much higher error rates (median Q-score ~ 28) than the plasmids under 3 kb. Since polishing provides large improvements for chromosomes, while having no decrease in accuracy for plasmids, we would recommend polishing all metagenomic assemblies.

### 4.4. Problem Isolates

We found that Raven and Redbean struggled to build assemblies of *E. coli* and *Salmonella enterica*. Latorre-Pérez et al. [11], also found that Raven and Redbean struggled with *E. coli* and *S. enterica* strains for the log and even mock communities from ZymoBIOMICS, both of which use the same *E. coli* and *S. enterica* strains as the HMW DNA Standard Mock Community. However, in a non-metagenomic study, Chen et al. [40] found that Raven could assemble complete genomes for a different strain of *E. coli* and possibly a different serovar of *Salmonella* (*S.* Typhimurium). This suggests that either the strain of *E. coli* used in the mock community is a problematic strain or that assembling genomes of *E. coli* combined with *S. enterica* is difficult. Breckell and Silander [13] found that strain specific characteristics of different *E. coli* made some *E. coli* strains harder to for assemblers to assemble, so it is possible that the strain of *E. coli* in the mock community could be a more difficult strain to assemble. However, Breckell and Silander [13] found that problematic strains of *E. coli* were problematic for all assemblers. Flye had few misassemblies for *E. coli* at 200× read depth and had more accurate assemblies of *E. coli* than Raven or Redbean. This evidence is not consistent with a problematic strain of *E. coli*. However, we cannot fully eliminate the idea that the strain of *E. coli* in the mock community may be a more difficult strain to assemble.

### 4.5. Other Studies

To the best of our knowledge, our study is the first study to compare metagenomic assemblies made by Flye, Raven, and Redbean using super-accurate basecalled reads. We found Flye still made more accurate and complete genomes than Raven or Redbean when used with highly accurate reads. This is consistent with a previous comparison of Flye, Raven, and Redbean assemblies made from the less accurate reads Latorre-Pérez et al. [11]. Like Sereika et al. [22] we found accurate genomes could be built from read depths as low as 30× using Flye (Q 45). This is an improvement from the Q-score of 43.6 at 80× read depth seen by Broddrick et al. [41] and the Q-score of around 40 seen by Latorre-Pérez et al. [11] for 6 Gb of data. We also know from Sereika et al. [22] that even higher accuracy can be achieved if a r10.4 flow cell is used instead of a r9.4 flow cell.

Like Latorre-Pérez et al. [11], we found that most community members had genome fractions over 99% at 200× read depth. However, our genome fractions for most community members were often at over 99.9%, which is higher than what Latorre-Pérez et al. [11] found at 3 Gb and 6 Gb. The only exceptions were assemblies of *E. coli* and *S. enterica* made by Raven and Redbean, which had lower genome fractions than what Latorre-Pérez et al. [11] found.

One key difference between this study and Latorre-Pérez et al. [11] is that the raw data used here had much longer reads (mean read N50 of 15 kb), where as Latorre-Pérez et al. [11] had 4 kb reads. This difference may account for the higher genome fractions for most community members, but does not explain the decrease in genome fractions for *E. coli* and *S. enterica* assemblies made by Raven or Redbean.

Like Breckell and Silander [13] and Latorre-Pérez et al. [11], we found Flye and Raven to be better than Redbean in assembling complete genomes. However, unlike Breckell and Silander [13], but like Latorre-Pérez et al. [11], we found Flye assembled more accurate assemblies than Raven. The difference may be that Breckell and Silander [13] looked at assembling single isolates instead of metagenomes in this study and Latorre-Pérez et al. [11]. This suggests that Raven may be better suited for assembling single isolates than metagenomics.

Like Wick and Holt [17], we found Flye needed more time and memory than Raven and Redbean to complete an assembly. The large time and memory demands of Flye may limit Flye to lab use or at least limit Flye to high end laptops. However, Flye was the only assembler able to assemble the entire mock community at Q-scores greater than 40. Furthermore, the use of the super-accuracy basecalling model will likely require a higher end laptop with a good GPU. This makes the high time and memory usage of Flye less of an issue.

## 5. Conclusions

We found Flye was more reliable than Raven or Redbean for building accurate and complete assemblies for both chromosomes and plasmids from metagenomic communities. We found that Raven and Redbean struggle to recover small plasmids. This suggests that Flye would be a better choice for assembling viral community members. For our study’s community, Raven and Redbean only performed better than Flye in the computational resources needed to build an assembly. However, for a metagenomic study using the superaccurate basecalling model, the extra time and memory usage needed to run Flye would likely be minimal. On the other hand, the cost in accuracy from problematic communities members or missing small plasmid and virus assemblies from Raven and Redbean could lead to misinterpretations. Thus, for future metagenomic studies that use the super-accurate basecalling model, we would recommend using Flye.

## Figures and Tables

**Table 1:** Community members metadata for the ZymoBIOMICS DNA Standard ^1^.

NRRL Accession	Organism	Plasmid ^2^	% GC	Genome Size (mb)	Gram	% Nucleotides	No. Genomes ^3^
B-354	*Bacillus subtilis*	0	43.9	4.045	+	14	13.20
B-537	*Enterococcus faecalis*	0	37.5	2.845	+	14	18.80
B-1109	*Escherichia coli*	1	46.7	4.875	-	14	10.90
B-33116	*Listeria monocytogenes*	0	38.0	2.992	+	14	17.80
B-3509	*Pseudomonas aeruginosa*	0	66.2	6.792	-	14	7.80
B-4212	*Salmonella enterica*	0	52.2	4.760	-	14	11.20
B-41012	*Staphylococcus aureus*	3	32.9	2.730	+	14	19.60
Y-567	*Saccharomyces cerevisiae*	NA	38.3	12.100	NA	2	0.63

^1^ Metadata information from https://files.zymoresearch.com/protocols/_d6322_zymobiomics_hmw_dna_standard.pdf, accessed on 15 July 2021. ^2^ Number of plasmids associated with organism. ^3^ No. genomes: genome copies per 100 genomes.

**Table 2:** Subsample statistics for each read depth. Each read depth had 12 subsamples.

Read Depth	Mean No. Reads	Mean Read N50	Mean Yield (mb)
Raw Data ^1^	3,538,810	14,703	31,073.91
10×	45,373	15,012.25	419.28
20×	91,345	15,020.75	838.87
30×	137,018	15,020.08	1258.72
50×	228,363	15,023.42	2099.35
100×	456,726	15,027.83	4198.77
200×	913,452	15,030.75	8398.78

^1^ initial data before subsampling.

**Figure A4:**
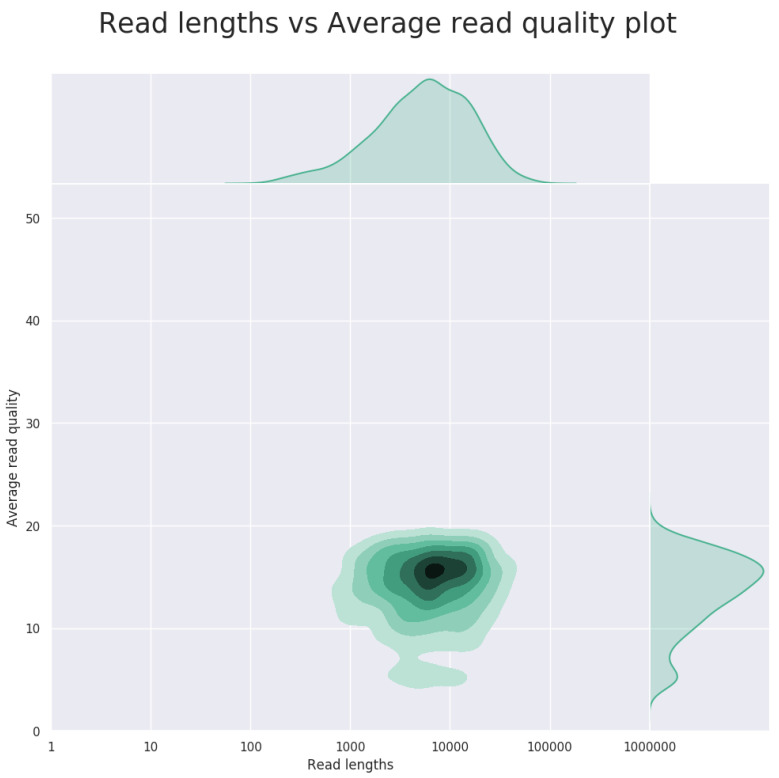
Histogram of read length and quality of unsubsampled data.

## Data Availability

The sequencing data for this project can be found in the NCBI SRA https://www.ncbi.nlm.nih.gov/sra/PRJNA903965 under accession number PRJNA903965.

## Appendix A

**Table A1:** Median genome fractions for all community members.

Assembler	Read Depth	*Staphylococcus*	*Salmonella*	*Pseudomonas*	*Listeria*	*Escherichia*	*Enterococcus*	*Bacillus*
Redbean	200	99.99	69.55	98	99.96	51.63	100	100
Raven	200	100	74.08	99.96	99.96	79.24	100	100
Flye	200	100	99.93	100	99.96	100	100	100
Redbean	100	99.97	71.70	97.81	99.92	53.10	99.31	99.91
Raven	100	100	68.57	99.93	99.96	80.50	100	100
Flye	100	100	99.93	100	99.96	100	100	100
Redbean	50	99.52	69.46	96.19	91.93	57.18	93.81	99.79
Raven	50	100	66.20	99.91	99.96	78.38	100	100
Flye	50	100	99.93	100	99.96	100	100	100
Redbean	30	98.66	68.20	97.45	95.05	57.96	98.69	99.58
Raven	30	100	73.96	99.94	99.96	77.76	99.99	100
Flye	30	100	99.93	100	99.96	100	100	100
Redbean	20	98.69	68.20	93.86	93.07	56.38	98.23	99.66
Raven	20	100	74.73	99.96	99.96	75.94	99.96	100
Flye	20	100	99.93	100	99.96	100	100	100
Redbean	10	99.67	67.83	97.35	95.05	56.97	97.03	99.56
Raven	10	100	79.44	84.94	99.96	74.66	94.18	93.23
Flye	10	100	99.93	100	99.96	100	100	99.95

## References

[1] Garmendía L., Hernández A., Sánchez M.B., Martínez J.L. (2012). Metagenomics and antibiotics. Clin. Microbiol. Infect..

[2] Petersen L.M., Martin I.W., Moschetti W.E., Kershaw C.M., Tsongalis G.J. (2019). Third-Generation Sequencing in the Clinical Laboratory: Exploring the Advantages and Challenges of Nanopore Sequencing. J. Clin. Microbiol..

[3] Bai G.H., Lin S.C., Hsu Y.H., Chen S.Y. (2022). The Human Virome: Viral Metagenomics, Relations with Human Diseases, and Therapeutic Applications. Viruses.

[4] Kumar N., Sharma S., Barua S., Tripathi B.N., Rouse B.T. (2018). Virological and Immunological Outcomes of Coinfections. Clin. Microbiol. Rev..

[5] Qin S., Ruan W., Yue H., Tang C., Zhou K., Zhang B. (2018). Viral communities associated with porcine respiratory disease complex in intensive commercial farms in Sichuan province, China. Sci. Rep..

[6] Goldstein S., Beka L., Graf J., Klassen J.L. (2018). Evaluation of strategies for the assembly of diverse bacterial genomes using MinION long-read sequencing. BMC Genom..

[7] Sevim V., Lee J., Egan R., Clum A., Hundley H.N., Lee J., Everroad R.C., Detweiler A.M., Bebout B.M., Pett-Ridge J. (2019). Shotgun metagenome data of a defined mock community using Oxford Nanopore, PacBio and Illumina technologies. Sci. Data.

[8] Amarasinghe S.L., Su S., Dong X., Zappia L., Ritchie M.E., Gouil Q. (2020). Opportunities and challenges in long-read sequencing data analysis. Genome Biol..

[9] Payne A., Holmes N., Rakyan V.K., Loose M.W. (2019). BulkVis: A graphical viewer for Oxford nanopore bulk FAST5 files. Bioinformatics.

[10] Jain M., Koren S., Miga K.H., Quic J., Rand A.C., Sasani T.A., Tyso J.R., Beggs A.D., Dilthey A.T., Fiddes I.T. (2018). Nanopore sequencing and assembly of a human genome with ultra-long reads. Nat. Biotechnol..

[11] Latorre-Pérez A., Villalba-Bermell P., Pascual J., Porcar M., Vilanova C. (2020). Assembly methods for nanopore-based metagenomic sequencing: A comparative study. Sci. Rep..

[12] Yang C., Chowdhury D., Zhang Z., Cheung W.K., Lu A., Bian Z., Zhang L. (2021). A review of computational tools for generating metagenome-assembled genomes from metagenomic sequencing data. Comput. Struct. Biotechnol. J..

[13] Breckell G.L., Silander O.K. (2021). Do You Want to Build a Genome? Benchmarking Hybrid Bacterial Genome Assembly Methods. bioRxiv.

[14] Chen Z., Erickson D.L., Meng J. (2020). Benchmarking hybrid assembly approaches for genomic analyses of bacterial pathogens using Illumina and Oxford Nanopore sequencing. BMC Genom..

[15] Kolmogorov M., Bickhart D.M., Behsaz B., Gurevich A.A.G., Rayko M., Shin S.B., Kuhn K.L., Yuan J., Polevikov E., Smith T.P.L. (2020). metaFlye: Scalable long-read metagenome assembly using repeat graphs. Nat. Methods.

[16] Kolmogorov M., Yuan J., Lin Y., Pevzner P.A. (2019). Assembly of long, error-prone reads using repeat graphs. Nat. Biotechnol.

[17] Wick R.R., Holt K.E. (2019). Benchmarking of long-read assemblers for prokaryote whole genome sequencing. F1000Research.

[18] Vaser R., Šikić M. (2021). Time and memory-efficient genome assembly with Raven. Nat. Comput. Sci..

[19] Ruan J., Li H. (2020). Fast and accurate long-read assembly with wtdbg2. Nat. Methods.

[20] Rizzi R., Beretta S., Patterson M., Pirola Y., Previtali M., Della Vedova G., Bonizzoni P. (2019). Overlap graphs and de Bruijn graphs: Data structures for de novo genome assembly in the big data era. Quant. Biol..

[21] Aniba M.R., Poch O., Thompson J.D. (2010). Issues in bioinformatics benchmarking: The case study of multiple sequence alignment. Nucleic Acids Res..

[22] Sereika M., Kirkegaard R.H., Karst S.M., Michaelsen T.Y., Sørensen E.A., Wollenberg R.D., Albertsen M. (2021). Oxford Nanopore R10.4 long-read sequencing enables near-perfect bacterial genomes from pure cultures and metagenomes without short-read or reference polishing. bioRxiv.

[23] Bokulich N.A., Rideout J.R., Mercurio W.G., Shiffer A., Wolfe B., Maurice C.F., Dutton R.J., Turnbaugh P.J., Knight R., Caporaso J.G. (2016). mockrobiota: A Public Resource for Microbiome Bioinformatics Benchmarking. mSystems.

[24] Wick R.R., Judd L.M., Cerdeira L.T., Hawkey J., Méric G., Vezina B., Wyres K.L., Holt K.E. (2021). Trycycler: Consensus long-read assemblies for bacterial genomes. Genome Biol..

[25] De Coster W., D’Hert S., Schultz D.T., Cruts M., Van Broeckhoven C. (2018). NanoPack: Visualizing and processing long-read sequencing data. Bioinformatics.

[26] Vaser R., Sović I., Nagarajan N., Šikić M. (2017). Fast and accurate de novo genome assembly from long uncorrected reads. Genome Res..

[27] Mikheenko A., Saveliev V., Gurevich A.A. (2016). MetaQUAST: Evaluation of metagenome assemblies. Bioinformatics.

[28] R Core Team (2015). R. A Language and Environment for Statistical Computing.

[29] Wilkinson L. (2011). ggplot2: Elegant Graphics for Data Analysis by WICKHAM, H. Biometrics.

[30] Wilke C.O. (2020). Cowplot: Streamlined Plot Theme and Plot Annotations for ’ggplot2’, R Package Version 1.1.1.

[31] Kassambara A. (2020). Ggpubr: ’ggplot2’ Based Publication Ready Plots, R Package Version 0.4.0.

[32] Wickham H. (2021). Tidyr: Tidy Messy Data, R Package Version 1.1.4.

[33] Dowle M., Srinivasan A. (2021). Data.table: Extension of ‘data.frame’, R Package Version 1.14.2.

[34] Wickham H. (2019). Stringr: Simple, Consistent Wrappers for Common String Operations, R Package Version 1.4.0.

[35] Neuwirth E. (2014). RColorBrewer: ColorBrewer Palettes, R Package Version 1.1-2.

[36] Gaudreault N.N., Madden D.W., Wilson W.C., Trujillo J.D., Richt J.A. (2020). African Swine Fever Virus: An Emerging DNA Arbovirus. Front. Vet. Sci..

[37] Kovalenko G., Ducluzeau A.L., Ishchenko L., Sushko M., Sapachova M., Rudova N., Solodiankin O., Gerilovych A., Dagdag R., Redlinger M. (2019). Complete Genome Sequence of a Virulent African Swine Fever Virus from a Domestic Pig in Ukraine. Microbiol. Resour. Announc..

[38] Breitbart M., Delwart E., Rosario K., Segalés J., Varsani A. (2017). ICTV Virus Taxonomy Profile: Circoviridae. J. Gen. Virol..

[39] Antipov D., Rayko M., Kolmogorov M., Pevzner P.A. (2022). viralFlye: Assembling viruses and identifying their hosts from long-read metagenomics data. Genome Biol..

[40] Chen Z., Erickson D.L., Meng J. (2020). Benchmarking Long-Read Assemblers for Genomic Analyses of Bacterial Pathogens Using Oxford Nanopore Sequencing. Int. J. Mol. Sci..

[41] Broddrick J.T., Szubin R., Norsigian C.J., Monk J.M., Palsson B.O., Parenteau M.N. (2020). High-Quality Genome-Scale Models From Error-Prone, Long-Read Assemblies. Front. Microbiol..

